# Indoor Test System for Liquid CO_2_ Phase Change Shock Wave Pressure with PVDF Sensors

**DOI:** 10.3390/s20082395

**Published:** 2020-04-23

**Authors:** Xing Huang, Qiyue Li, Xin’ao Wei, Xiaoxiao Yang, Dayou Luo, Haideng Zeng, Hongwei Wang

**Affiliations:** 1 School of Resources and Safety Engineering, Central South University, Changsha 410083, China; xinghuang92@csu.edu.cn (X.H.); wxa2017@csu.edu.cn (X.W.); 195512141@csu.edu.cn (X.Y.); hdZeng@csu.edu.cn (H.Z.); ww0609@csu.edu.cn (H.W.); 2 Department of Civil and Environmental Engineering, Francis College of Engineering, University of Massachusetts Lowell, Lowell, MA 01854, USA; Dayou_Luo@student.uml.edu

**Keywords:** PVDF sensors, test system, liquid CO_2_ phase change, shock wave pressure test

## Abstract

Liquid carbon dioxide phase change fracturing technology (LCPCFT) has been widely used in engineering blasting due to the advantage of no flames, and no toxic and harmful gas. However, few studies have been conducted on the acquisition of shock wave pressure and its loading characteristics, which are key parameters in fracturing. Referring to the CO_2_ in-situ fracturing technology, an indoor test system for shock wave pressure generated during LCPCFT has been built, with a protected polyvinylidene fluoride (PVDF) piezoelectric sensor. Then three verification experiments with different radial distances between the fracturing tube and test points were carried out on the test system, and in each experiment, four PVDF sensors as four test points were arranged with different axial distance from the detonating point to test the pressure distribution. The experimental results show that when the radial distance between the fracturing tube and test points is not too large (≤345 mm), the pressure generated during LCPCFT is approximately uniformly distributed within the axial length of the fracturing tube, but when it is relatively large (≈895 mm), the results between different test points are in a certain degree of dispersion. And finally, this paper uses the intraclass correlation coefficient (ICC) and coefficient of variation (C_V_) of peak pressure and impulse to process the test results to evaluate the reliability and stability of the test system. Evaluation results show that the test results are in good consistency. The test system in this paper has good stability and high reliability. The test system provides a useful tool for accurately obtaining the shock wave pressure, which is helpful for further research on LCPCFT.

## 1. Introduction

Liquid carbon dioxide phase change fracturing technology (LCPCFT) is a technology that uses a chemical agent to heat and vaporize liquid CO_2_ stored in a steel tube, causing the liquid CO_2_ to undergo a phase change in a short time and generate huge expansion pressure to break the rock. This technology was firstly developed and used by a British company, Cardox, in 1914, mainly to solve practical engineering problems in coal mining. Because LCPCFT has the advantages of small blasting vibration, no flame, and no toxic and harmful gas in the process of fracturing [1], it has been widely used in the fields of engineering blasting with complex construction conditions and high blasting requirements, such as coal seam permeability enhancement [2], surface soil stripping [3], tunnel excavation [4] and subway construction [5].

Hu [6] conducted permeability enhancement experiments of a coal seam by using LCPCFT; the test results show that cracks generated inside the coal seam will greatly increase the gas permeability of the coal seam. In addition, the process of LCPCFT is endothermic, which belongs to cold blasting, so the process of permeability enhancement is safer and more environmentally friendly. Because the small surface tension of supercritical CO_2_, it can penetrate into the interior of rock mass along the cracks, therefore the rock failure is accompanied by the generation of tensile cracks [7], and its rock breaking ability is usually better than that of a water jet [8]. Under the effect of competitive adsorption, the gaseous CO_2_ produced during the fracturing process will convert the gas absorbed in the coal seam to a free state, which will increase the amount of gas released and reduce the possibility of a gas outburst during excavation [9]. Through experiments and numerical simulations, Sun [10] believed that the rock breaking under LCPCFT is the result of a stress wave and gasified CO_2_,and the influence of initial confining pressure and blasting pressure on the effect of LCPCFT are also analyzed [11]. Zhu [12] analyzed the damage evolution process of concrete specimens under the action of high-pressure gas explosion through numerical simulation, and established its damage model.

At present, the research on LCPCFT usually focuses on the direct fracturing effect or its practical engineering application. The research on obtaining the shock wave pressure and its loading characteristics during the fracturing process is relatively rare. However, the shock wave pressure first acts on the borehole wall, and then propagates in the rock as a stress wave. The pressure and its loading characteristics are one of the key parameters that determine its fracture effect. Therefore accurate measurement of its value is of great significance for the study of LCPCFT.

Compared with piezoelectric ceramics and quartz sensors, polyvinylidene fluoride (PVDF) piezoelectric sensors have the advantages of light weight, good flexibility, high sensitivity, and wide frequency bandwidth. More importantly, PVDF can be processed into any shape without its piezoelectric capability degraded [13], which is convenient for installation. Therefore, since Bauer [14] demonstrated the use of polarization technology to improve the repeatability of stress measurements and obtained stable PVDF piezoelectric film successfully, the sensor has been widely used in impact load testing [15,16,17], and has shown good test performance. K. Murata [18] tested the detonation pressure of non-ideal explosives with a PVDF sensor. The test results show that a PVDF sensor is an effective tool to measure the detonation pressure of non-ideal explosives in a long reaction zone. Boteler, J.M. [19] tested the internal stress history of polymer composite with an embedded PVDF sensor, and the results are in good agreement with the theoretical calculations. In an underwater explosion shock wave pressure test, Paul Gustavson [20] affixed PVDF sensors to the front and back of the structure: the PVDF on the front tested the stress and strain caused by the explosion shock wave, while the back only monitored the strain signal. Then the two test results were superimposed to eliminate the strain effect of the PVDF sensor. The test results are basically consistent with the theoretical calculations.

This paper takes the acquisition of shock wave pressure and its loading characteristics, which are the most basic research angles of LCPCFT, as the research goal. Firstly, referring to the CO_2_ in-situ fracturing technology, an indoor liquid CO_2_ phase change shock wave pressure test system was constructed independently, based on the protected PVDF sensor. Then three verification experiments with different radial distances between the fracturing tube and test points were carried out on the test system, and in each experiment, four PVDF sensors as four test points were arranged with different axial distances from the detonating point, to test the pressure distribution. Finally, the intraclass correlation coefficient (ICC) and coefficient of variation (C_V_) of the test results are calculated to evaluate the stability and reliability of the test system.

## 2. Test System Design

**Figure 1:**
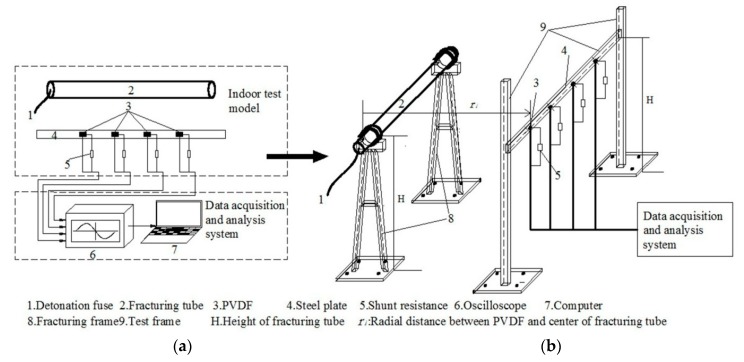
(**a**) Test system; (**b**) Indoor test model.

With reference to the CO_2_ in-situ fracturing technology, an indoor test system for CO_2_ phase change shock wave pressure was designed; it was mainly composed of two parts: (1) an indoor test model, and (2) a data acquisition and analysis system. As shown in Figure 1a, a fracturing tube is filled with liquid CO_2_. After firing the detonation fuse, a large amount of thermal energy is generated by the chemical agent in the heating tube. After liquid CO_2_ in the tube absorbs heat, the pressure and temperature rise rapidly, then a great pressure is generated on the inner wall of the tube. When the pressure is greater than the failure strength of the steel tube, it ruptures, and high-pressure gaseous CO_2_ is ejected; then a shock wave is formed. When the shock wave reaches the steel plate after it propagates in the air, it impacts the surface of the polyvinylidene fluoride (PVDF) piezoelectric sensor. The varying shock wave pressure generates a piezoelectric signal in the sensor, and then converts the collected piezoelectric signal into a pressure signal, with mathematical calculation, to obtain the pressure versus time curve of a shock wave pressure on the surface of the steel plate.

### 2.1. Indoor Test Model

This part is mainly composed of four parts: model building, PVDF sensor design, test circuit design, and PVDF protection and calibration.

#### 2.1.1. Model Building

The purpose of this system is to test the pressure and loading characteristics of shock wave pressure on the borehole wall during the use of liquid carbon dioxide phase change fracturing technology (LCPCFT). Because the field experiment is usually destructive, its repeatability is poor, and the field conditions such as terrain and coupled electric fields are complicated; the experiment conditions are difficult to control. Therefore, referring to the CO_2_ in-situ fracturing technology, an indoor liquid CO_2_ phase change shock wave pressure test model is built, as shown in Figure 1b.

**Figure 2:**
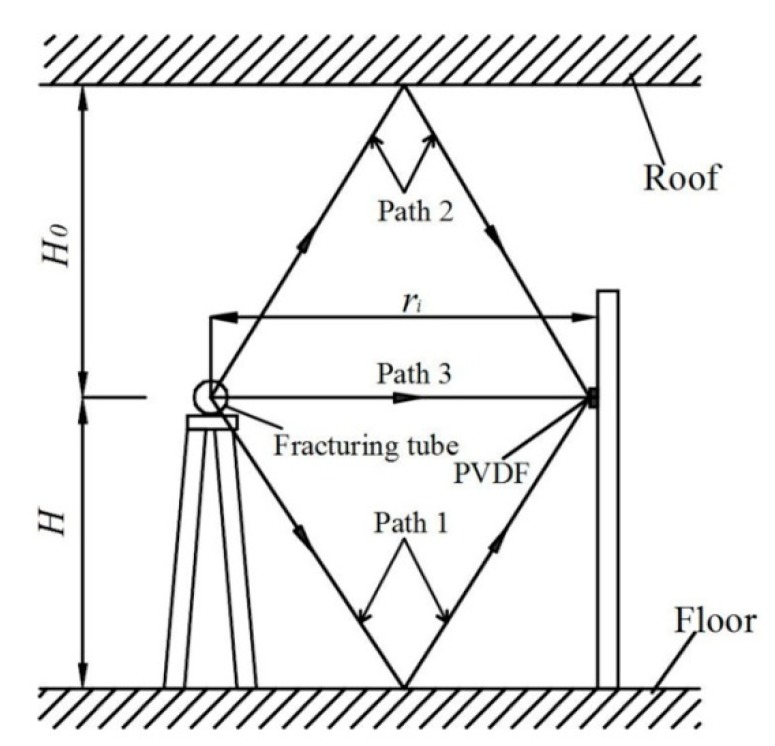
Schematic diagram of shock wave propagation path.

The fracturing tube filled with a certain quantity of liquid CO_2_ is placed horizontally on the fracturing frame with a height of H above the ground, which is the same as the PVDF installed on steel plate. During acquisition period, PVDF should not be interfered with by the reflected waves from the ground and roof. As shown in Figure 2, the time of shock wave propagating to PVDF sensor after reflected from the ground (path 1) or roof (path 2) should be longer than that of the shock wave propagating directly to PVDF (path 3) plus the time of signal acquisition. Assuming that the velocity of the shock wave is constant during propagation, H should be:

(1) $$2\sqrt{H^{2}+\frac{r_{i}^{2}}{4}}\geq r_{i}+t_{0}v$$

(2) $$2\sqrt{{H_{0}}^{2}+\frac{r_{i}^{2}}{4}}\geq r_{i}+t_{0}v,$$

where H_0_ is the distance between the roof and fracturing tube, the total height of laboratory is 4.5 m, H_0_ = 4.5 − H; t_0_ is the signal acquisition time of PVDF, which is usually within 200 μs according to the previous experiments; *v* is the propagation velocity of the shock wave in the air, because the larger *v* is, the larger H and H_0_ are, and the model is more stringent. The detonation wave velocity of explosives, which is usually larger than the velocity described in this paper, is generally about 3000 m/s, so it is assumed that *v* = 3000 m/s in this paper; *r_i_* is the axial distance between the center of fracturing tube and PVDF, the experiments in this paper are 0.895, 0.345, and 0.1 m, respectively. From Equations (1) and (2), this paper obtains 0.60 m ≤ H ≤ 3.90 m, and takes H = 1.5 m in the model.

The steel plate is installed at the same height as the center of the fracturing tube, with an axial distance of *r_i_*; the length of the steel plate is 1.2 m, the width is 4 cm, and the thickness is 2 cm. It is affixed, connected with two parallel steel columns by nuts to reduce the influence of vibration. The PVDF piezoelectric sensor is affixed to the steel plate during the test.

In actual engineering, the CO_2_ fracturing tube is usually placed in the borehole. Under the constraint of the rock near the borehole, the rock is broken by the shock wave pressure. The following approximations are mainly performed during the establishment of the model:

1. In this model, it is actually designed to measure the shock wave pressure on the surface of steel plate; in actual engineering, it is the pressure on the wall of borehole. According to the Rankine-Hugoniot equations, when the stress wave reaches the interface of different media and incident vertically, the transmission and reflection stresses at the interface are given by the Equations (3) and (4) [21]. Because rock is a natural material, its composition is complex, the properties of different rocks are very different, and it is not convenient to process. The wave impedance of rock and steel plate are both much larger than that of air. With Equations (3) and (4), it can be seen that the transmission stresses of the two should be approximately equal, therefore this approximation is reasonable.
  (3) $$\sigma_{r}=\frac{\rho_{2}c_{p2}-\rho_{1}c_{p1}}{\rho_{2}c_{p2}+\rho_{1}c_{p1}}\sigma_{i}$$
  (4) $$\sigma_{t}=\frac{2\rho_{2}c_{p2}}{\rho_{2}c_{p2}+\rho_{1}c_{p1}}\sigma_{i},$$
  where *σ_i_* is the incident stress, ρ_1_, c_p1_, ρ_2_, and c_p2_ are the medium density and wave velocity before and after the incident, respectively, and the product of the two are the wave impedance of the medium before and after the incident, respectively.
2. In actual engineering, the shock wave pressure is usually constrained by the rocks near the borehole wall, but the indoor model is used to test the shock wave pressure in the free field, which is a simplification of the complex constraint conditions in the field test, so as to facilitate the repetitive comparative test. The main purpose of this paper is to establish a reasonable and reliable shock wave pressure test method, and the constraints will not affect it too much, so the approximation is reasonable to a certain extent.

#### 2.1.2. PVDF Sensor Design

After the gasification CO_2_ causes damage to the fracturing tube, a shock wave is formed and propagates in the air. When the wavefront curvature radius R is much larger than the size of the sensor’s sensitive element L, the shock wave can be regarded as a plane wave for the sensor, and then it can test the shock wave pressure relatively accurately. R and L need to satisfy the relationship as follows [22]:

(5) $$\frac{L}{R}\leq \frac{1}{5}.$$

**Figure 3:**
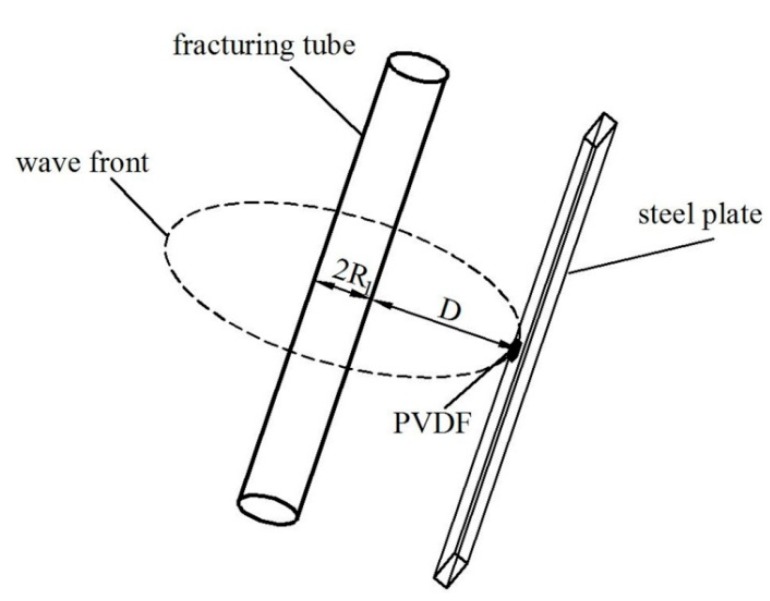
Schematic diagram of shock wave propagation.

The fracturing tube used in this paper is a cylindrical steel tube with a diameter of 90 mm, a length of 1 m, and the ratio of height to diameter is relatively large: 11.11. Therefore, it is assumed that the shock wave propagates two-dimensionally in a plane perpendicular to the axis of the fracturing tube. Because the shock wave propagates in a uniform air medium before reaching the test mechanism, as shown in Figure 1b and Figure 2, it is assumed that the shock wave propagates with the fracturing tube axial direction as a symmetry axis, as shown in Figure 3. Therefore, *R* can be expressed as:

(6) $$R=R_{1}+D$$

where *R*_1_ is the radius of the fracturing tube, which is 0.045 m, and *D* is the propagation distance in the air. For the three experiments carried out in this paper, the propagation distances are 0.85, 0.3, and 0.055 m respectively.

With the Equations (5) and (6), L should not be more than 20.0 mm. Due to the disturbance of fragment splashing on the shock wave propagation and other factors, the shock wave’s propagation is not strictly two-dimensional symmetrical, so it must be reduced on the basis of the upper limit size of the sensitive element. In this paper, a PVDF sensor with a square sensitive element whose side length is 10 mm is used for testing.

PVDF sensors and steel plates are two media with different wave impedances. Shock waves will interfere when passing through different media, and the thinner the thickness, the less obvious the interference effect [23]. According to Li [24], the polytetrafluoroethylene (PTFE) protective device with thickness less than 0.5 mm will not significantly interfere with the tested explosion pressure. In order to reduce the transmission and reflection of the shock wave at the interface between the PVDF and the steel plate, the total thickness of the whole PVDF sensor assembly installed on the steel plate is set to less than 0.25 mm. The thickness of the PVDF in this paper is 0.05 mm.

#### 2.1.3. Test Circuit Design 

The PVDF sensor generates charge under the shock wave pressure. The test circuit is connected to an oscilloscope to record the amount of charge generated during the test and then converted into a pressure signal with Equation (7):

(7) Q=AKσ

where *Q* is the cumulative charge generated during the test, σ is the stress acting on the per unit area of PVDF, *A* is the area of the sensor, and *K* is the piezoelectric constant of the sensor.

The test circuit is mainly used to test the cumulative charge *Q* generated during the experiment. At present, the commonly used test circuits mainly include current mode and charge mode. The charge mode is used to connect the sensor in parallel with a matching capacitor, and the output charge of PVDF is obtained by measuring the voltage of the capacitor. In the current mode, the sensor and a matching resistor are connected in parallel to form a test circuit; the charge generated during the experiment is released through the resistor, and then the current signal on the resistor is monitored with the oscilloscope. The current signal is calculated to obtain the corresponding pressure signal. In this paper, the current mode is chosen as the test circuit for the following reasons:

1. Because current is defined as the rate of change of charge versus time, *i = dQ/dt*, and charge is linearly related to stress (as in Equation (7)), the current mode actually measures the rate of change of stress *dσ/dt*. For the high loading rate of CO_2_ phase change shock wave pressure, the method can represent more transient information during the loading process, and is very sensitive to the structure of shock wave. However, the charge mode is suitable for measuring pressure signals with a long pulse rise time [20].
2. In order to avoid damage to the oscilloscope caused by cracked tube fragments during the test, the length of the signal cable is generally more than 5 m, and the reactance of the signal cable in the charge mode has a greater interference on the capacitance.

**Figure 4:**
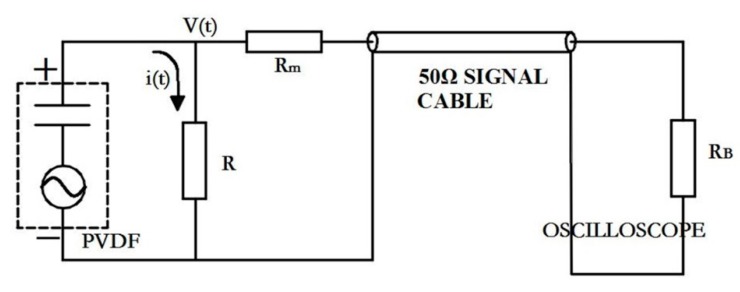
Test circuit.

Therefore, the test circuit is designed as shown in Figure 4. The PVDF piezoelectric sensor is connected in parallel with the resistor *R*. The voltage on the parallel resistor R is tested to obtain its transient current *i*(*t*), and then the charge *Q*(*t*) is obtained by numerical integration. In Figure 4, *R_m_* is the matching resistance. The characteristic impedance of the signal cable is 50 Ω. In order to match the impedance of the test circuit, *R_m_* + *R* = 50 Ω, *R_B_* is the input impedance of the oscilloscope, and *R_B_* > 1 MΩ. Because *R_B_ >> R_m_ + R*, the shunt effect of the oscilloscope can be ignored, and it can be obtained that:

(8) $$i\left(t\right)=\frac{V\left(t\right)}{R}$$

(9) $$Q\left(t\right)=\int_{0}^{t}\frac{V\left(t\right)}{R}dt.$$

In Figure 4, *R_m_* not only plays the role of impedance matching, but also reduces the voltage on parallel resistance R, which plays the effect of voltage division. Obviously, the value of *R_m_* has an influence on the signal peak. In order to set the sensitivity of the oscilloscope reasonably, there must be an estimated value for the expected peak signal before the test. If the peak value of the signal is not accurately estimated, it may cause signal clipping or only occupy a small part of the range, resulting in an increase in test errors. Therefore the resistance values of *R_m_* and *R* need to be determined.

Assuming the loading rate of shock wave pressure is constant during the pulse rises, the rise time is *t*_1_, the maximum stress is *σ_max_*, and the peak voltage signal generated is *V_max_*, then according to Equations (8) and (9), the peak voltage can be obtained [25]:

(10) $$V_{max}=\frac{QR}{t_{1}}=\frac{A\sigma_{max}KR}{t_{1}}$$

It was found in previous experiments that under the test conditions in this paper, the pulse rise time is generally greater than 15 μs, the peak pressure is generally less than 600 MPa, the piezoelectric constant *K* = 47.5 pC/N, *A* = 100 mm^2^, when *R* = 50 Ω, according to Equation (10) the peak voltage *V_max_* = 9.5 V, and the range of the oscilloscope is ±10 V, which does not exceed the oscilloscope range. To simplify the circuit, set *R_m_* = 0, and the parallel resistance *R* = 50 Ω.

#### 2.1.4. Protection of PVDF

**Figure 5:**
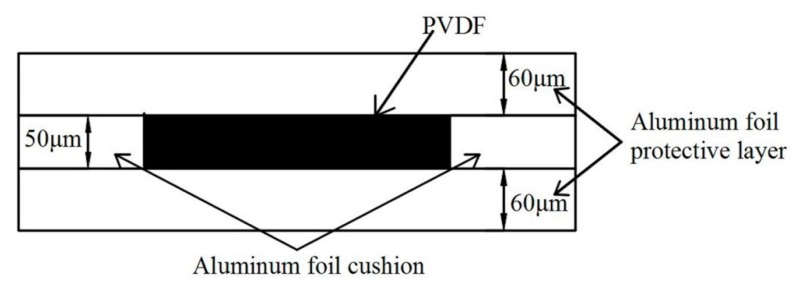
PVDF sensor assembly.

PVDF is a kind of piezoelectric polymer material with a certain flexibility. The material is fragile, and easy to be destroyed under the impact of external shock wave pressure, resulting in the distortion of measured data. In addition to the piezoelectric effect, PVDF sensors also have pyroelectric effect; a change in temperature will also cause the PVDF sensor to generate charge, and form extra current in the circuit. In this paper, 60 μm thick aluminum foil tape is pasted on both sides of the PVDF for protection and thermal insulation. In order to avoid the deformation and stress concentration of the PVDF sensor assembly caused by the peripheral gaps around PVDF, since the thickness of aluminum foil is approximately the same as that of PVDF, aluminum foil is also used to flatten the PVDF sensor assembly, as shown in Figure 5.

**Figure 6:**
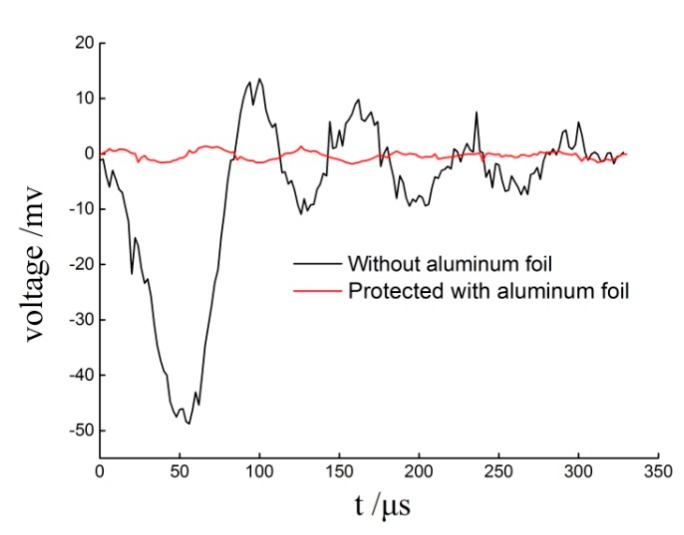
Thermal insulation effect of aluminum foil.

In order to examine the thermal insulation effect of aluminum foil, the exposed PVDF and the protected PVDF are respectively placed in hot water at 100 °C, and the voltage versus time curve is obtained, as shown in Figure 6. The results show that the protected PVDF does not produce an obvious electrical signal within 300 μs, and the thermal insulation effect of aluminum foil is good.

#### 2.1.5. Dynamic Calibration of PVDF

The use of aluminum foil will affect the piezoelectric constant of PVDF, and then affect the test results. Therefore, before the shock wave pressure test, the protected PVDF sensor needs to be calibrated to determine its piezoelectric constant. In this paper, the dynamic calibration tests are conducted on the split Hopkinson pressure bar (SHPB). During calibration, two identical PVDF sensors are sandwiched between the incident bar and the transmission bar, and the average value of the signals measured by the two sensors is used for data processing.

**Figure 7:**
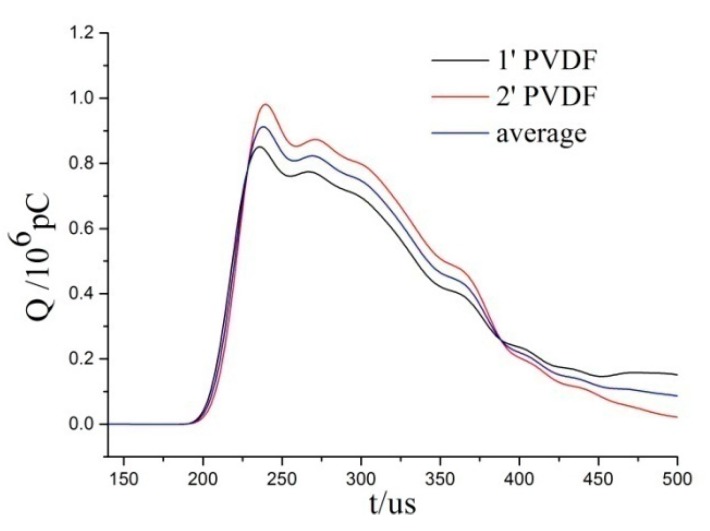
Typical curves of calibration test.

**Figure 8:**
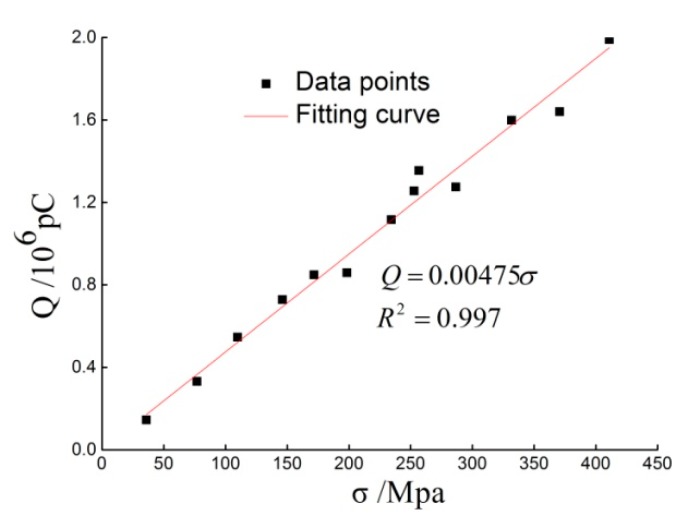
Calibration curve of protected PVDF by aluminum foil.

A total of 14 impact tests were performed; 13 effective PVDF charge versus time curves are obtained, and Figure 7 is a typical one. The charge amount and stress obtained from the calibration test are linearly fitted as shown in Figure 8. The coefficient of determination (R^2^) is 0.997, with good fitting effect. According to the fitting result, the piezoelectric constant of PVDF protected with aluminum foil *K* = 47.5 pC/N, slightly higher than the 43.94 pC/N provided by the manufacturer.

### 2.2. Data Analysis and Acquisition System

The oscilloscope collects the shock wave pressure signal generated during the experiment through the test circuit. The selection of the oscilloscope has a great influence on the quality of the collected signal, which mainly includes four parameters: bandwidth, sampling rate, record length, and voltage sensitivity [25].

1. Bandwidth: in general, to collect accurate test signals, the oscilloscope bandwidth should be more than three to five times that of the signal bandwidth. The relationship between the bandwidth *f_B_* (MHz) and the pulse rise time *t*_1_ (μs) is [26]:
  (11) $$f_{B}t_{1}\approx 0.35$$
  According to previous experiments, the pulse rise time under the test conditions in this paper is generally more than 15 μs, so the signal bandwidth is less than 233 KHz. If calculated five times, the oscilloscope bandwidth should be greater than 1165 KHz. This paper chooses the NUXI-1004 oscilloscope produced by Sichuan Tuopu Measurement & Control Technology Co., Ltd. (Sichuan, China), with a bandwidth of 2.5 MHz, which meets the test requirements.
2. Sampling rate: according to the Nyquist sampling theorem, in order to restore the original signal information without distortion, the sampling frequency should be more than twice the bandwidth of the measured signal. The maximum sampling rate of the oscilloscope in this paper is 10 MHz, which meets the test requirements.
3. Record length: this parameter reflects the ability of the oscilloscope to continuously acquire and store sampling points at the highest sampling rate. Record length = sampling time * sampling rate. In this paper, the oscilloscope owns four channels, and record length of each one is 256 MS. Under the maximum sampling rate of 10 MHz, the maximum continuous sampling time of the oscilloscope is 25.6 s, and the total test time is usually within a few seconds, which meets the test requirements.
4. Voltage sensitivity: in order to reduce the measurement error, the voltage sensitivity of the oscilloscope must be set properly to match the test signal. The range of the oscilloscope in this paper has four types: ±1, ±2, ±5 and ±10. Therefore, the peak voltage under the test condition should be estimated with Equation (10) before test, and then the appropriate range of the oscilloscope should be selected.

## 3. Experimental Arrangement

In this paper, polyvinylidene fluoride (PVDF) calibrated with a split Hopkinson pressure bar (SHPB) is used as the sensor to test the shock wave pressure of the liquid CO_2_ phase change. The inner and outer diameters of the fracturing tube are 78.4 mm and 90 mm, respectively, and the length is 1.0 m. The mass of CO_2_ filled inside is about 3.8 kg. The aluminum foil for PVDF protection should be pasted tightly and flattened to avoid stress concentration. The steel plate should be tested with flatness measuring equipment before installation to ensure that the surface is smooth and flat. To ensure firm adhesion, all components should be cleaned thoroughly with tissues and swabs wetted with isopropanol before pasting PVDF, and then apply epoxy resin evenly on the aluminum foil. Then paste it on the surface of the steel plate, and press the components with a cotton swab to ensure that the epoxy resin joined with no bubbles. After installation, the total thickness of the whole PVDF sensor assembly is tested with a digital vernier caliper, and the results are generally between 200 and 250 μm. If the thickness is greater than 250 μm, it should be removed and reinstalled.

The experiments are all carried out indoors, with a distance of more than 5 m from the surrounding walls, 1.5 m from the ground and 3 m from the roof. The test conditions can be approximately regarded as an unconstrained free field. The test temperature is generally around 20 °C, the state of the filled CO_2_ is generally the same. All fracturing tube used in the test are produced by Hunan Junkai Non-Explosive Blasting Science & Technology Co., Ltd. (Hunan, China), in the same batch.

**Figure 9:**
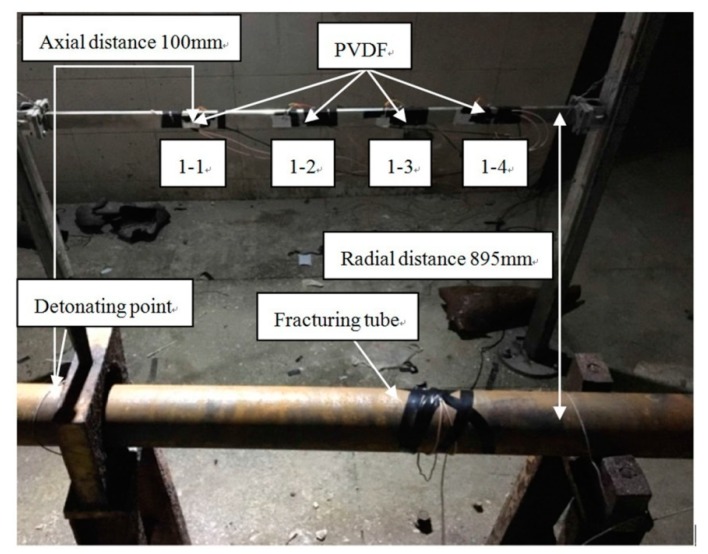
Layout of experiment 1.

In this paper, three experiments are carried out, and four test points with different axial distance from the detonating point are arranged in each experiment. The experimental arrangement is shown in Table 1. In the table, the axial distance refers to the distance between the test point and the detonating point, and the radial distance refers to the distance between the test point and the center of the fracturing tube. Experiment 1 is shown as Figure 9. The numbers of PVDF in three experiments are 1-1–1-4, 2-1–2-4 and 3-1–3-4, respectively.

## 4. Test Results

**Figure 10:**
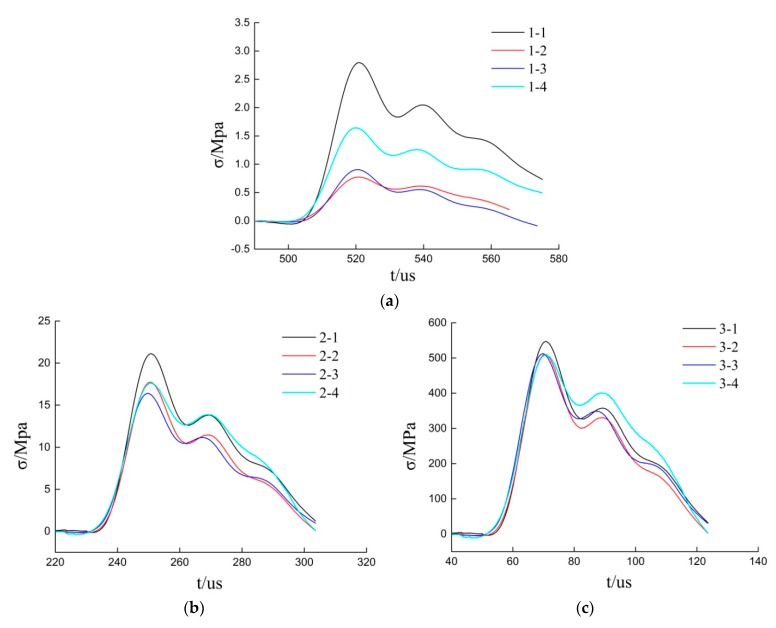
Experiment results: (**a**) Pressure versus time curves for experiment 1; (**b**) Curves for experiment 2; (**c**) Curves for experiment.

During the experiment, the shock wave pressure acts on the PVDF sensor, and the generated voltage signal is stored in the oscilloscope through the test circuit, then Equations (7)–(9) are used to calculate the shock wave pressure versus time curves. The pressure versus time curves for experiments 1, 2, and 3 are shown in Figure 10a−c.

Because the destruction ability of high pressure gas is mainly determined by peak pressure and impulse [27,28], they are also one of the most critical parameters obtained through the test system. The impulse is calculated as follows:

(12) $$I=\int_{0}^{t}\sigma \left(t\right)dt$$

where, *I* is the impulse of shock wave, *t* is the acting time, and *σ*(*t*) is the acting pressure.

The difference between test results of four test points in experiment 1 is relatively high, probably due to the long distance of the shock wave propagation, obviously interrupted by uncontrollable factors, such as the attenuation of shock wave and fragments of fracturing tube. In experiments 2 and 3, which are closer to the fracturing tube, the differences in peak pressure and impulse are relatively small: the maximum and minimum peak pressures in experiment 2 are measured by PVDF 2-1 and 2-3, respectively, at 21.12 MPa and 16.40 MPa and the variation range is 29% of the minimum peak pressure. In experiment 3, the maximum and minimum peak pressures are measured by PVDF 3-1 and 3-4, respectively, at 546.91 MPa and 509.55 MPa, and the variation range is 4% of the minimum peak pressure. The impulse distribution is similar to the peak pressure, as shown in Table 2, in which the peak pressure and impulse of the four measuring points in three experiments are listed in detail. With Table 2 and Figure 10, it can be concluded that when the distance between the test point and the fracturing tube is not too large (≤345 mm), the shock wave impulse, peak pressure, and overall trend of pressure versus time curves are all not much different. At this time, the axial distance from the detonating point will not significantly affect the pressure distribution of CO_2_ phase change shock wave.

The reason for this phenomenon is that before the experiment, the liquid CO_2_ is uniformly filled in the fracturing tube; at this time, the system is in an equilibrium state, and the internal pressure is equal everywhere. Under the excitation of the energy of the heating tube, the liquid CO_2_ gasifies and expands instantly, and the pressure and temperature increase. The gasified CO_2_ is constrained by the fracturing tube radially, and the generated pressure will inevitably transfer to the axial direction. The gas expansion and temperature rise will speed up the convection speed of the internal CO_2_, further readjust the internal pressure of the system and will tend to be evenly distributed. The system is in a quasi-static state before the fracturing tube is damaged, and its internal pressure is approximately equal everywhere. After the fracturing tube is broken, it is difficult to continue to produce a larger impact pressure due to the free expansion of CO_2_ in the air. Therefore, although the detonation point is located at one end of the fracturing tube, according to the experimental results, when the distance between the test point and the fracturing tube is not too large (≤345 mm), under the experimental conditions of this paper, the pressure generated during the experiment is approximately uniformly distributed within the axial length of the fracturing tube. The assumption in Section 2.1 that the phase change shock wave of CO_2_ propagates in two-dimensional symmetry is reasonable to some extent. However, when the distance is relatively large (≈895 mm), due to the large propagation distance, the peak pressure and impulse of each test point are obviously different under the interference of shock wave attenuation, fracturing tube fragments and other factors. In fact, in actual engineering, the uncoupling coefficient is usually less than 4, and in this paper, the radius of the fracturing tube is 45 mm, so the distance between the borehole wall and the center of the fracturing tube is usually less than 180 mm. If the constraints in actual engineering are ignored, 345 mm is large enough.

## 5. Discussion

For such a newly developed test system, the overall performance should be evaluated to ensure that the test results of the test system have a certain degree of reliability. According to Hopkins [29], for the evaluation of the stability and reliability of a device or system, a typical error and re-test reliability should be calculated. In general, in order to facilitate the comparison of different conditions, the typical error needs to be normalized, that is, divided by the average value of the results and converted into the coefficient of variation (C_V_). The larger the C_V_ value is, the lower the stability is; the re-test reliability is evaluated by the intraclass correlation coefficient (ICC).

With the conclusion in Section 4, when the distance between the test point and the fracturing tube is not too large, the shock wave propagates symmetrically in two dimensions, and the pressure is evenly distributed within the axial length of the fracturing tube. Therefore, the C_V_ of peak pressure and impulse obtained from four test points in each experiment are calculated to evaluate the stability of the test system, and the ICC of the test results is used to evaluate the re-test reliability. The test results of experiment 1 are also processed in the same way. ICC (two-way mixed effects, single measurements, consistency) is calculated as Equation (13) [30,31], with 95% confidence intervals (CI). ICC values are interpreted to define the degree of re-test reliability as: <0.50 = poor; 0.50–0.75 = moderate; 0.75–0.90 = good; and > 0.90 = excellent [32]. C_V_ are interpreted to define the degree of stability as [33]: <0.10 = strong; 0.1–1.0 = moderate; and >1.0 = weak. Details are shown as Table 3.

(13) $$ICC=\frac{MS_{R}-MS_{E}}{MS_{R}+\left(K-1\right)MS_{E}}$$

where *MS_E_* is the mean square error and *MS_R_* is the mean square for rows.

As shown in Table 3, results obtained at different test points in each experiment are in good consistency. The ICC values of the test results of experiments 2 and 3 are 0.979 and 0.988, respectively. The C_V_ of peak pressure and impulse are 0.111 and 0.113 (experiment 2), and 0.035 and 0.069 (experiment 3), respectively. When the distance between the test point and the center of fracturing tube is not too large (≤345 mm), re-test reliability of the test system is excellent, stability of peak pressure and impulse is strong or nearly strong. In experiment 1, ICC is 0.695, and C_V_ for peak pressure and impulse are 0.609, 0.674, respectively. Although influenced by fragments and shock wave attenuation, re-test reliability and stability are still moderate. The test system in this paper has good stability and high reliability.

## 6. Conclusions

In this paper, referring to the CO_2_ in-situ fracturing technology, an indoor test system for shock wave pressure produced during the liquid carbon dioxide phase change fracturing technology (LCPCFT) has been built, and the influence of polyvinylidene fluoride (PVDF) size, PVDF protection and calibration, the test circuit, and oscilloscope parameters on the test results are also analyzed. 

Based on the test system, three verification experiments are performed, and the experimental results show that when the distance between the fracturing tube and test points is not too large (≤345 mm), the pressure generated during the experiment is approximately uniformly distributed within the axial length of the fracturing tube. However, when the distance is relatively large (≈ 895 mm), the results between different test points are in a certain degree of dispersion.

Finally, the intraclass correlation coefficient (ICC) is used to evaluate the re-test reliability of the test system, and coefficient of variation (C_V_) for peak pressure and impulse are used to evaluate stability. The evaluation results show that the ICC for experiments 2 and 3 are 0.979 and 0.988, respectively; retest reliability is excellent, and the stability of peak pressure and impulse are strong or nearly strong. Although disturbed by a large distance between the fracturing tube and test points, the re-test reliability and stability in experiment 1 are still moderate. The test system in this paper has good stability and high reliability.

The successful establishment of the test system will help the further study of LCPCFT, such as the prediction of damage effect, which will enhance the engineering application value of the technology.

## Figures and Tables

**Table 1:** Arrangement of test points.

Number of Experiment	Number of Test Point	Axial Distance/mm	Radial Distance/mm
1	1-1	100	895
	1-2	300	
	1-3	500	
	1-4	700	
2	2-1	100	345
	2-2	300	
	2-3	500	
	2-4	700	
3	3-1	100	100
	3-2	300	
	3-3	500	
	3-4	700	

**Table 2:** Impulse and peak pressure of each test point.

Test Point Number Experiment Number		1	2	3	4
1	Peak pressure/MPa	2.80	0.77	0.90	1.65
	Pulse /Pa*s	111.56	29.35	26.74	69.36
2	Peak pressure /MPa	21.12	17.72	16.40	17.63
	Pulse /Pa*s	713.41	579.54	570.05	681.02
3	Peak pressure /MPa	546.91	510.25	512.26	509.55
	Pulse /Pa*s	18,475.79	16,690.73	17,802.85	19,680.95

**Table 3:** Stability and reliability evaluation of test results.

Experiment Number	Re-Test Reliability		Typical Error C_V_	
	ICC	95%CI	Peak Pressure	Impulse
1	0.695	0.667–0.722	0.609	0.674
2	0.979	0.976–0.981	0.111	0.113
3	0.988	0.986–0.989	0.035	0.069
